# Understanding Host-Pathogen Interactions in Acute Chorioamnionitis Through the Use of Animal Models

**DOI:** 10.3389/fcimb.2021.709309

**Published:** 2021-07-27

**Authors:** Amanda Brosius Lutz, Salwan Al-Nasiry, Boris W. Kramer, Martin Mueller

**Affiliations:** ^1^Department of Obstetrics and Gynecology, Inselspital, Bern University Hospital, Department for BioMedical Research (DBMR), University of Bern, Bern, Switzerland; ^2^Department of Obstetrics and Gynecology, GROW School of Oncology and Developmental Biology, Maastricht University Medical Centre (MUMC), Maastricht, Netherlands; ^3^Department of Pediatrics, Maastricht University Medical Centre (MUMC), Maastricht, Netherlands

**Keywords:** chorioamnionitis, preterm birth, animal models, host-pathogen interaction, pregnancy

## Abstract

Inflammation of the chorion and/or amnion during pregnancy is called chorioamnionitis. Acute chorioamnionitis is implicated in approximately 40% of preterm births and has wide-ranging implications for the mother, fetus, and newborn. Large disease burden and lack of therapeutic approaches drive the discovery programs to define and test targets to tackle chorioamnionitis. Central to the advancement of these studies is the use of animal models. These models are necessary to deepen our understanding of basic mechanisms of host-pathogen interactions central to chorioamnionitis disease pathogenesis. Models of chorioamnionitis have been developed in numerous species, including mice, rabbits, sheep, and non-human primates. The various models present an array of strategies for initiating an inflammatory response and unique opportunities for studying its downstream consequences for mother, fetus, or newborn. In this review, we present a discussion of the key features of human chorioamnionitis followed by evaluation of currently available animal models in light of these features and consideration of how these models can be best applied to tackle outstanding questions in the field.

## Introduction

### What Is Chorioamnionitis?

Chorioamnionitis is strictly defined by the accumulation of inflammatory cells at the chorion and amnion. The etiology of inflammation is in most cases infectious, although a role for sterile inflammation due to tissue injury (e.g. hypoxia) is increasingly appreciated. In practice, the term chorioamnionitis often refers to involvement of additional maternal (decidua, maternal circulation) and fetal (placental villi, umbilical cord, amniotic fluid, fetal circulation) compartments beyond the amnion and chorion to which the inflammatory process spreads during disease (Kim et al., 2015a). Histologically chorioamnionitis belongs to one of the four major patterns of placental injury and can be further characterized into acute, sub-acute, and chronic forms corresponding to the accumulation of neutrophils, lymphocytes, or both, respectively (Kim et al., 2015b; Redline et al., 2021). Acute chorioamnionitis is the best-studied of these entities and is characterized by maternal neutrophils (from decidual venules) migrating into the chorion and amnion in response to (microbial) chemotactic factors in the amniotic fluid (maternal inflammatory response) (Redline et al., 2021). Later, fetal neutrophils join the response by migrating across large vessel walls in the chorionic plate and umbilical cord (fetal inflammatory response). The grading indicates the severity of the inflammatory responses while staging reflects their duration (Redline et al., 2021). For example, the clinically relevant high-grade fetal inflammatory response is defined by confluent neutrophils and evidence of endothelial damage, myocyte disarray, or mural thrombi in chorionic vessels. Except in cases where invasive testing (example: amniocentesis) is performed, the diagnosis of chorioamnionitis is assigned to patients with clinical signs suggestive of inflammation at the maternal/fetal interface and remains speculative until histologic confirmation. This statement is especially true of sub-acute and chronic chorioamnionitis, which are less commonly associated with overtly abnormal clinical findings and likely highly underdiagnosed in locations where placental histology is not routinely performed (Cappelletti et al., 2020). Understanding the prevalence and the short and long-term clinical significance of acute, sub-acute, and chronic chorioamnionitis is an area of active and important research. For this review, we focus on the host-pathogen interactions of acute chorioamnionitis (called chorioamnionitis from here on) and its implications for maternal and fetal health. We will review the acute chorioamnionitis in light of animal models, which are necessary to understand pathophysiology of this syndrome or develop novel treatments.

### What Is the Disease Burden of Acute Chorioamnionitis and What Are Current Therapeutic Limitations?

Chorioamnionitis complicates as many as 40–70% of spontaneous preterm births and 1–13% of term births (Tita and Andrews, 2010). The etiology of adverse fetal/neonatal outcomes is multifactorial with inflammatory processes being a crucial driving force overlayed on critical windows of fetal development. Associated outcomes include bronchopulmonary dysplasia (BPD), necrotizing enterocolitis (NEC), retinopathy of prematurity (ROP), intracerebral hemorrhage (ICH) and periventricular leukomalacia (PVL). On the maternal side, women suffering from chorioamnionitis have a two to three times higher risk for cesarean delivery and a three to four times greater risk for endomyometritis, wound infection, pelvic abscess, bacteremia, and postpartum hemorrhage (Chapman et al., 2014). Not surprisingly, understanding of basic mechanisms of host-pathogen interactions central to chorioamnionitis disease pathogenesis is a priority of perinatal medicine.

The development of prophylactic (as well as therapeutic) strategies to prevent and/or modulate chorioamnionitis is the common goal. Multiple approaches have been considered but most of them with limited success. Studies evaluating the association of vaginal microbiota and preterm premature rupture of the membranes (PPROM) point towards optimization of the vaginal microbiome as a prophylactic strategy (Brown et al., 2019; Singer et al., 2019). Thus far, targeting the vaginal microbiome has not been successful in preventing premature birth and new approaches, such as modulation of the maternal immune response are increasingly in focus (Al-Nasiry et al., 2020). These strategies target immune modulation such as a decrease in pro-inflammatory cytokine production rather than antibiotic treatments (Mueller et al., 2014; Di Simone et al., 2017; Spinelli et al., 2020; Xiong and Wintermark, 2020). Although promising, these therapies have not yet been put into use clinically. Additional strategies are based on invasive detection of intra-amniotic inflammation or early clinical diagnosis of chorioamnionitis with subsequent therapy (Chapman et al., 2014; Yoon et al., 2019; Yeo et al., 2021). These prenatal approaches to chorioamnionitis include intrapartum antibiotic treatment and prenatal corticosteroid and MgSO_4_ prophylaxis (Xiong and Wintermark, 2020). Of these, especially the use of antibiotics in cases of PPROM is supported by randomized controlled trials in patients with chorioamnionitis. Besides antibiotic treatment, postnatal (neonatal) therapeutic approaches currently under study include dietary modification and therapeutic hypothermia (Kainonen et al., 2013). In general, each of the therapeutic strategies just discussed targets a specific aspect of the chorioamnionitis disease process with relevance to a specific disease outcome. Efficacy thus far leaves the majority of the disease burden untouched. Not only are randomized controlled trials studying the efficacy of existing therapies and combinations thereof imperative to improving disease outcomes, extensive study is desperately needed to better understand the basic mechanisms underlying chorioamnionitis so that strategies for disease prevention can be better targeted and optimized.

### The Way Forward: Animal Models of Chorioamnionitis

The large disease burden of chorioamnionitis and the lack of therapeutic approaches drive the discovery programs to uncover and test prevention and treatment targets. Central to the advancement of these studies is the development and use of animal models to deepen our understanding of disease pathogenesis. This need for good disease models of chorioamnionitis is especially pressing given the challenges of performing clinical trials and obtaining cell and tissue samples during pregnancy and the neonatal period.

Models of chorioamnionitis have been developed in numerous species, including mice, rabbits, sheep, and non-human primates. The different models present a range of strategies for initiating an inflammatory response, varying technical challenges, and varying degrees of translational relevance. Due to the heterogeneity of outcomes of human chorioamnionitis no disease model fully recapitulates the human scenario, underscoring the importance of scrutiny when choosing a model. The most appropriate animal model for studying aspects of microbial spread or the maternal immune response may, for example, not at all be the best choice for investigation of long-term consequences for the exposed fetus (Lee et al., 2011; Vlassaks et al., 2012). With careful choice of the model best-suited to answer a well-defined scientific question, animal models have enormous potential to advance our understanding of the host-pathogen interactions of chorioamnionitis disease pathogenesis.

In the present review, we will focus on the use of animal models to study chorioamnionitis and its associated perinatal outcomes. As with any disease, the process of modeling chorioamnionitis in animals is iterative. Early models are based on known pillars of human disease. The relevance of findings yielded from the animal model is then tested in humans. If human disease relevance is confirmed these findings add to our knowledge of the human disease and allow for more refined studies in the corresponding animal models. Along these lines, we begin in this review with a summary of known key features of human acute chorioamnionitis. With these pillars in mind, we discuss existing animal models of chorioamnionitis, recent insights gained using these models, and areas for future investigation using animal models of the disease (van Well et al., 2017).

### The Anatomy of the Human Maternal-Fetal Interface (Morphology Defines the Function)

**Figure 1:**
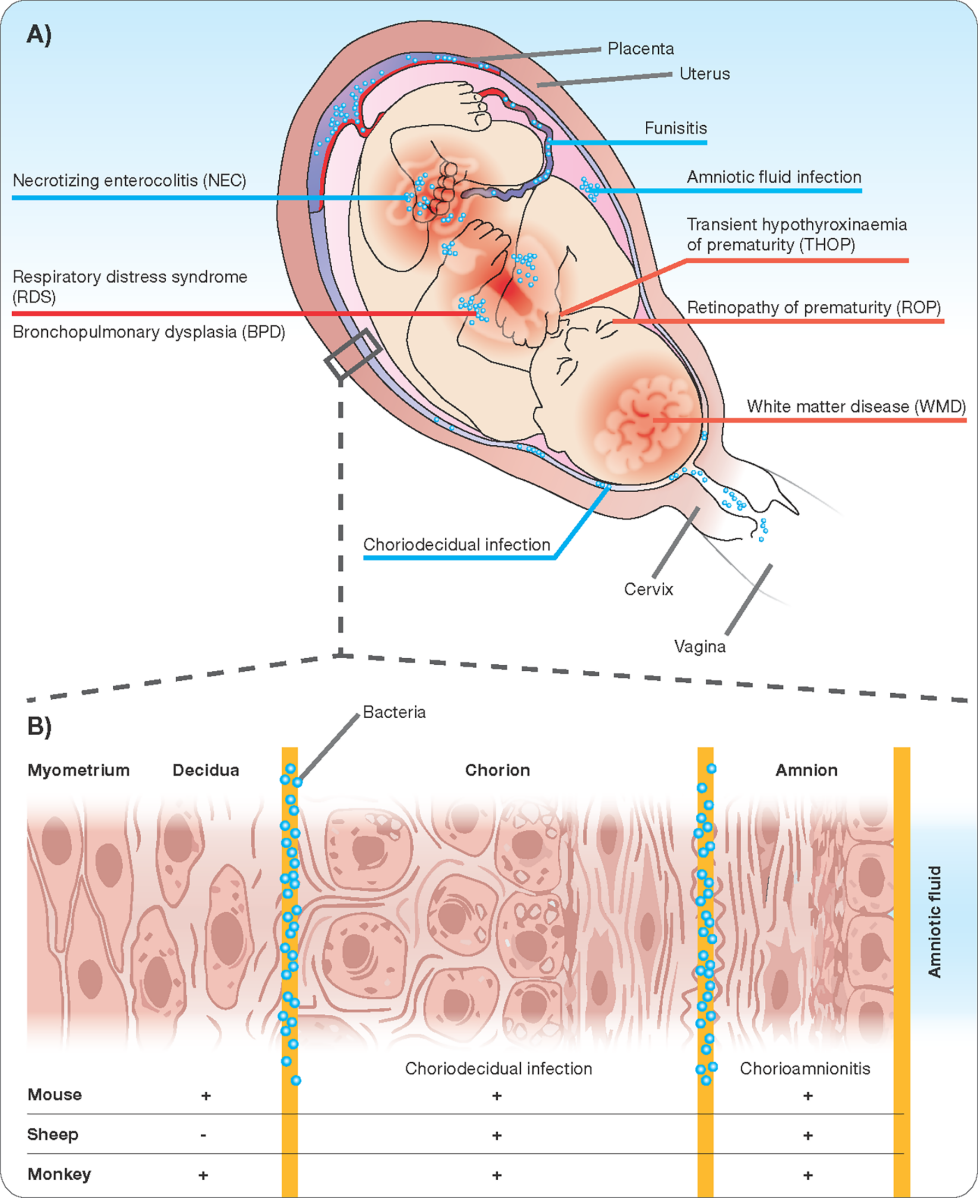
Chorioamnionitis as a multiorgan disease of the fetus **(A)** with potential sites of infection/inflammation **(B)**.

The human maternal-fetal interface is composed of three major layers. Beginning on the maternal side is the decidua, the endometrium of the pregnant uterus, followed by the chorion and the amnion or fetal membranes, which in early development are formed from and continuous with the embryonic endoderm, mesoderm, and ectoderm. In extraplacental regions, these three layers are in close vicinity. The placenta forms a disc-like specialization of the decidua on one side and chorion on the other allowing the interface of villous penetrations of fetal vessels with maternal blood brought into the intervillous space by maternal spiral arteries (Carter, 2016; Hafez, 2017). This anatomic arrangement is referred to as “hemo-chorial”. Fetal vessels surrounded by Wharton’s Jelly enter and exit the placenta through the umbilical cord, which is itself ensheathed in a continuation of the chorion and amnion. Not surprisingly Wharton`s Jelly are an excellent source of early mesenchymal stem cells (Mueller et al., 2016). Each compartment of the interface has a unique cellular composition creating compartment-specific immune interactions that influence the likelihood of bacterial invasion (Cappelletti et al., 2020). Finally, amniotic fluid fills the space between the developing fetus and the amnion. It begins as an ultrafiltrate of maternal blood and later in gestation is produced through fetal urination and fetal lung fluid. Despite the constant debate whether amniotic fluid is sterile (Al-Nasiry et al., 2020), sterile intra-amniotic inflammation and the following immune response in the chorioamniotic membranes is increasingly being recognized as contributor to the preterm labor and birth immunobiology (Romero et al., 2014; Romero et al., 2015). Inflammation may occur in each of the compartments described above (**Figure 1**). Strictly speaking, chorioamnionitis refers to inflammation within the amnion and/or chorion, and under physiological conditions neutrophils are rarely seen in the chorioamniotic membranes (Gomez-Lopez et al., 2017; Tong et al., 2019). However, neutrophils are rapidly recruited to sites of infection (following chemoattractants) and once they infiltrate the villous tree of the placenta, the term acute villitis is used. If neutrophils infiltrate the structures of the umbilical cord (artery, vein, or surrounding Wharton’s Jelly), the term acute funisitis is used (Kim et al., 2015a). Intraamniotic inflammation occurs when neutrophils migrate into the amniotic cavity and can be detected in the amniotic fluid. A spectrum of outcomes ensues depending on the degree of inflammation, maternal inflammatory response, and gestational age of the pregnancy. Not surprisingly, chorioamnionitis is considered increasingly as a syndrome and defining key features is essential.

## Defining Key Features of Human Chorioamnionitis

### Feature 1: Ascending Infection

Data on causative infectious agents come from culture and molecular analysis of amniotic fluid, cordocentesis, and neonatal blood. These studies reveal that the most probable route of infection is ascending invasion from the lower genital tract (Romero et al., 2019). The most routinely isolated organisms include genital mycoplasmas, Group B Streptococcus, Gardnerella vaginalis, Fusobacteria, and E.coli. Fungi, including candida species, have also been reported. Increasingly sensitive detection techniques challenge the long held notion that the healthy intrauterine environment is sterile and physiologic amniotic fluid bacterial colonization is presently debated. (Stinson et al., 2019). These methods also add new organisms to the generally accepted list of perpetrators, some of which have yet to be characterized (DiGiulio et al., 2010). To date, no association between bacterial presence in amniotic fluid and increased risk of preterm birth in patients without clinical chorioamnionitis at the time of amniotic fluid sampling has been established (Aagaard et al., 2014; Lim et al., 2018; Stinson et al., 2020). Whether changes in the bacterial communities of the lower genital tract allow for a clear identification of women at risk of progression to chorioamnionitis is an active area of current research and multicenter prospective randomized trials to assess the therapeutic potential of prophylactic alteration of the vaginal microbiome are lacking (Vinturache et al., 2016; Al-Nasiry et al., 2020). So far, evidence connecting the structure of the vaginal microbial community to the timing of delivery is limited. Increasing data suggests that an increase in the diversity of the vaginal microbial community is associated with an increased risk of preterm delivery but the sampling methods used need to be carefully considered (Stout et al., 2017; Elovitz et al., 2019; Fettweis et al., 2019).

Less commonly, infection arrives at the membranes through hematogenous spread (Kim et al., 2015a; Al-Nasiry et al., 2020). In these cases, the placenta serves as both a physical and immunological barrier that effectively blocks most infectious agents from entering the fetal circulation and amniotic fluid. However, selected organisms exhibit tropism for placental cell types and can overcome this defense (depending again on gestational age) and cause chorioamnionitis through invasion of the placenta from the maternal bloodstream. These agents include Listeria monocytogenes, Zika virus, Cytomegalovirus, Plasmodium species, and toxoplasmosis, syphilis (Treponema pallidum), varicella-zoster virus, parvovirus B19, Rubella virus, human immunodeficiency virus, or herpes virus infections. Mechanisms of spread across the placental barrier are incompletely understood and warrant future investigation (Arora et al., 2017).

### Feature 2: Primary Intrauterine Inflammation

The very arrival of infectious agents in the amniotic cavity is the initiating event for the inflammatory process that characterizes chorioamnionitis. In the case of ascending infection, current understanding holds that bacteria migrate first through the cervix across the decidua and then across the chorion and amnion into the amniotic fluid (**Figure 1**). The intra-amniotic inflammatory response is coordinated by cells of the chorion and amnion, fetal skin cells, and the umbilical cord, all of which respond through toll-like receptor activation to pathogen-associated molecular patterns (PAMPs) by secreting pro-inflammatory cytokines such as TNF-α and IL-1β, and IL-6 (Cappelletti et al., 2020). In response to the resulting cytokine gradient, maternal neutrophils migrate out of decidual vessels and into the fetal membranes in addition to fetal neutrophils migrating out of fetal umbilical vessels into surrounding umbilical cord tissue, constituting funisitis (Kim et al., 2015a). Recent evidence points towards the potential of antibiotic administration in patients at risk of preterm birth and with intact membranes (Yoon et al., 2019; Yeo et al., 2021). This feature of primary intrauterine inflammation is in contrast to the scenario in which maternal systemic inflammation precedes intrauterine inflammation. An example of such a scenario is maternal pyelonephritis or pneumonia. In these situations, an association with preterm birth is recognized, but the intermediate mechanisms linking maternal systemic inflammation to preterm birth are not yet clear and whether chorioamnionitis is a necessary intermediate is unknown but probable.

### Feature 3: Polymicrobial Infection, Biofilms, and Maternal Immune Modulation (Two-Hit Model and the Concept of Viral Priming)

When an infectious agent of chorioamnionitis is identified, in most cases a single agent is isolated. However, approximately 30% of amniotic fluid samples demonstrate the growth of more than one organism (DiGiulio et al., 2010; Payne and Bayatibojakhi, 2014). Work in recent years has also observed that rather than a planktonic distribution, intra-amniotic infections in some cases form biofilms, visible *via* ultrasound study as sludge within the amniotic cavity (Espinoza et al., 2005; Romero et al., 2008). The organization of bacteria in biofilms may help to explain the resistance of intraamniotic infections to maternal antibiotic treatments (DiGiulio et al., 2008; DiGiulio et al., 2010). The frequent observation of biofilms within the amniotic fluid without immediate progression to preterm rupture of membranes suggests that a second hit may be required in some instances to activate a final common pathway of inflammation within fetal tissues. Along these lines, a role for viral priming has been suggested in which some viruses such as influenza do not directly cause inflammation at the maternal-fetal interface but modulate the maternal immune system to be less tolerant of existing or upcoming bacterial challenge (Romero et al., 2008; Kim et al., 2015a; Cross et al., 2017). Other examples include local changes such as the cervical susceptibility to the following insult (Racicot et al., 2013). The ongoing COVID-19 pandemic has already highlighted the risk for adverse pregnancy outcomes in pregnant women with COVID-19 infection (Walker et al., 2020). Finally, the maternal immune system is known to undergo modulation in order to tolerate the presence of fetal antigens during gestation. Whether variations in this immune modulation could underlie individual differences in susceptibility to microbial invasion or production of inflammation is a fascinating area of ongoing research (Firmal et al., 2020). It opens the possibility to maternal prophylactic treatments as well (Di Simone et al., 2017; Spinelli et al., 2020).

### Feature 4: Fetal Inflammatory Response and Finally Preterm Birth

The presence of infection within the amniotic cavity opens the door for progression to fetal invasion. Routes of entry into the fetus include the respiratory tract, gastrointestinal tract, skin, ear, and conjunctiva. From here, infection and inflammation can remain local, as in the case of isolated neonatal pneumonitis or dermatitis, or may progress to systemic fetal inflammation (Kim et al., 2015a). Fetal Inflammatory Response Syndrome (FIRS) is defined as an elevated fetal plasma concentration of the cytokine IL-6, a major mediator of the acute phase response (Kallapur et al., 2014; Jung et al., 2020). The presence of FIRS is associated with a shorter interval to delivery, increased neonatal morbidity (after adjustment for gestational age at birth), and injury to multiple fetal organs (**Figure 1**) (Jung et al., 2020). In addition to chemokines and cytokines, bacterial invasion of the amniotic cavity stimulates the production of other inflammatory mediators such as prostaglandins, reactive oxygen radicals, and proteases (Kallapur et al., 2011). These products initiate myometrial contractility, cervical ripening, and induce membrane rupture, together increasing the likelihood of preterm birth (Menon et al., 2020). Intriguingly, as mentioned above, extra-uterine maternal infections, such as pyelonephritis, and pneumonia are also associated with spontaneous preterm birth in the absence of evidence for the hematogenous spread of infection to the maternal-fetal interface. The mechanisms involved have yet to be completely elucidated, but likely involve systemic inflammation and immune modulation (Kim et al., 2015a).

## Animal Models of Acute Chorioamnionitis

### General Implications for Modeling in Animals: Anatomy and Immune Composition

While no animal model recapitulates the human condition perfectly, mechanistic studies in animal models are crucial and a necessity for allowing initial hypothesis testing before confirmation in humans (Kallapur et al., 2011). The ongoing experiments in animal models of chorioamnionitis fall loosely into one of three categories: 1. Studies aimed to understand consequences of chorioamnionitis for fetal and neonatal health, 2. Studies aimed to understand the mechanisms of chorioamnionitis-induced preterm birth, and 3. Studies aimed to understand initiating events leading to inflammation at the fetal-maternal interface. Given that no model recapitulates all aspects of human chorioamnionitis, the decision, which animal model to use, must be guided by the scientific question asked. The histologic structure of the placenta, the number of fetuses, the host microbiology and immunology, the gestation, and the size of the fetus are all important aspects to consider (Lambermont et al., 2014; Wolfs et al., 2014; Schmidt et al., 2016).

### Rodents

Rodents are the most often used species in chorioamnionitis research due to their hematochorial placenta (closely resembling human placental architecture), short gestation time, the potential for genetic modification, and relative affordability. The mice models are fairly well suited to projects aimed at understanding mechanisms of bacterial spread and preterm birth as germ-free environments/caging is possible (Seferovic et al., 2019). These models provide further insights into the development of materno-fetal immunity and therapeutics (Garcia-Flores et al., 2018; Gomez-Lopez et al., 2018; Ganal-Vonarburg et al., 2020; Spinelli et al., 2020). The effects of prenatal treatments can be visualized in crucial organs like the placenta or the fetal brain and correlated to basic functional outcomes (Di Simone et al., 2017; Spinelli et al., 2020). Differences in immune system components and the consequences of being raised in facilities devoid of microbial diversity should be kept in mind, given the central role of host immune responses in acute chorioamnionitis pathophysiology (Tao and Reese, 2017). In general, murine models of chorioamnionitis are widely used and accepted.

### Non-Rodents (Sheep, Non-Human Primates)

In terms of fetal development and physiology, sheep match human development much more closely than rodents and are a better choice for studies aimed at understanding the fetal inflammatory response and its short and long-term implications for fetal organ development and function (Bieghs et al., 2010; Vlassaks et al., 2012; Kemp et al., 2016; Gussenhoven et al., 2017; Hafez, 2017; Gussenhoven et al., 2018). Ovine models allow the manipulation of the fetus and sophisticated surgeries of different epithelial organs like lung, brain, or gastrointestinal (Moss et al., 2003; Kramer et al., 2010; Maneenil et al., 2015; Nikiforou et al., 2016), On the maternal side, however, significant differences between ovine and human membrane and placental anatomy preclude the use of these models for studies of preterm birth (Kadyrov et al., 2007; Hafez, 2017). Non-human primates are the closest approximation of maternal and fetal physiology, but cost, labor-intensive husbandry, limited tools for manipulation, and ethical restrictions limit their use for initial work examining basic mechanisms. Despite these limitation, non-human primates are extremely valuable to decipher preterm labor pathology or test novel treatment regimens (Vadillo-Ortega et al., 2002; Sadowsky et al., 2003; Sadowsky et al., 2006; Gravett et al., 2007; Grigsby et al., 2012). Above we summarized several key features of human chorioamnionitis that, despite ongoing work and many gaps in our understanding, are areas of established or growing consensus. We will now discuss each of these features in the context of existing animal models of chorioamnionitis. In **Table 1**, we attempt to compare the models by evaluating each one according to these predefined criteria.

**Table 1:** Summary of characteristics of different animal models of acute chorioamnionitis.

Species	Model (Agent, route of infection)	Ascending infection	Histologic or molecular inflammation at maternal-fetal interface^1^	Systemic maternal inflammation or clinical signs thereof	Fetal inflammatory response^2^	Preterm Birth	Selection of Reference(s)
Human acute chorioamnionitis		+	+	-/+	+	+	
Murine models							
	*Group B Streptococcus*, vaginal infection	+	+	?^3^	?^3^	?	(Randis et al., 2014)
	Live *E.coli*, vaginal infection	+	?	?	?	+	(Akgul et al., 2014)
	Heat-killed *E.coli*, uterine horn injection (mini laparotomy)	–	+	?	?	+	(Filipovich et al., 2009; Chin et al., 2016)
	Influenza A, intranasal	–	–	?	?	?	(Chaturvedi et al., 2015)
	*Listeria monocytogenes*, i.v.	–	+	?	?	?	(Chaturvedi et al., 2015)
	Synthetic lipopeptides (TLR ligands), i.p.	–	?	+	?	+/-^4^	(Cappelletti et al., 2018)
	LPS, US guided intra-amniotic injection	–	?	–	?	+	(Gomez-Lopez et al., 2018)
	LPS, US-guided intrauterine injection	–	+	+^5^	?	+	(Rinaldi et al., 2015)
	Double hit model: virus/PolyI:C/Lm; LPS, i.p	–	+	+	?	+	(Racicot et al., 2013; Cappelletti et al., 2017; Cross et al., 2017)
	LPS, i.p.	–	+	+^5^	+	+	(Akgul et al., 2014; Chin et al., 2016; Gomez-Lopez et al., 2018; Spinelli et al., 2020)
	*E.coli*,/LPS, intracervical	–	+	?	?	+	(Reznikov et al., 1999)
	LPS, intrauterine injection (mini laparotomy)	–	+	+/-^5,6^	?^7^	+	(Elovitz et al., 2011; Migale et al., 2015; Gomez-Lopez et al., 2018)
Sheep models							
	US-guided PAMCysK4, poly I:C dsRNA	–	?	?	?	?	(Hillman et al., 2008)
	US-guided Il-a injection	–	+	?	?	?	(Willet et al., 2002; Berry et al., 2011)
	US-guided intra-amniotic LPS, instrumented fetuses	–	?	?	+	–	(Kemp et al., 2014; Garcia-Flores et al., 2018)
	US-guided intra-amniotic Ureaplasma parvum	–	+	?	?	–	(Hillman et al., 2008; Kallapur et al., 2011; Miura et al., 2014)
Non-human primate models							
	US-guided intra-amniotic Il1-B injection	–	+	+	+	?	(Vadillo-Ortega et al., 2002; Sadowsky et al., 2003; Kallapur et al., 2013)
	US-guided Intra-amniotic Ureaplasma, instrumented animals	–	+	–	?	+	(Novy et al., 2009) (Grigsby et al., 2012)
	US-guided Intra-amniotic Ureaplasma, non-instrumented animals	–	–	–	–	?	(Senthamaraikannan et al., 2016)
	US-guided Intra-amniotic LPS	–	+	–	+	?	(Schmidt et al., 2016; Presicce et al., 2018)
	*group B Streptococcus*, instrumented animals	+	+	+	+	+	(Gravett et al., 2007)
	US-guided intra-amniotic Il1-B, TNFα, Il-6, Il-8 injection	–	+	+	+	+	(Sadowsky et al., 2006)

?not reported or unclear from reported data.

^1^Decidua or fetal membranes.

^2^Elevated concentration of IL-6 in fetal circulation.

^3^Positive maternal and fetal blood cultures, but circulating cytokines not examined.

^4^Variable result depending on specific lipopeptide used.

^5^Maternal hypothermia in Gomez-Lopez et al.

^6^Dose dependent.

^7^IL-6 only in fetal brain examined in Migale et al. + present. - absent.

### Feature 1: Ascending Infection

For studies aimed at understanding mechanisms of ascending spread, there are several models of chorioamnionitis involving vaginal inoculation of bacteria such as GBS and E. coli to mimic an ascending route of infection (Akgul et al., 2014; Randis et al., 2014). Unlike the human vaginal mucosa, the superficial layers of the murine vaginal epithelium are heavily keratinized, raising the possibility that bacterial adherence and spread within this compartment may differ in comparison with the human vaginal mucosa. Differences in vaginal pH, hormone metabolism and cycling, and microbiota may also influence the spread of inoculated organisms (Randis et al., 2014; Al-Nasiry et al., 2020). Nevertheless, these models demonstrate robust placental inflammation, allowing for their use in understanding specific factors in the pathogenesis of ascending infection (**Table 1**).

Inoculating agents used in animal models of acute chorioamnionitis include live bacteria and viruses as well as heat-killed bacterial and viral and bacterial PAMPs (Regan et al., 2016) that are recognized by host toll-like receptors (TLRs) in multiple organs such as lipopolysaccharides (LPS) and Poly I:C (Hillman et al., 2008; Filipovich et al., 2009; Mueller et al., 2014; Cappelletti et al., 2018). LPS is an endotoxin found in the cell wall of gram-negative organisms and activates the innate immune response by binding primarily TLR 4 (Berry et al., 2011; Kallapur et al., 2011). LPS is well suited for studies of inflammatory responses of fetal brain or lung as endotoxin dose and time differences can be precisely controlled (Kramer et al., 2001; Spinelli et al., 2020). However, the organisms most often isolated from the amniotic fluid are not gram-negative bacteria. Ureaplasma and mycoplasma, both wall-less bacteria, are known to activate the innate immune response through TLR 2, 6, and 9 and are the most common bacteria isolated in chorioamnionitis (Collins et al., 2013; Wolfs et al., 2013). The intra-amniotic infection with ureaplasma and their contribution to neonatal morbidity/mortality is increasingly recognized (Senthamaraikannan et al., 2016; Motomura et al., 2020). While the final common pathway of immune system activation leading to preterm delivery may look the same whether initially stimulated by gram-positive or negative bacteria, these differences in initiating stimulus should not be overlooked. Further studies focused on understanding the early stages of the maternal immune response considering the types of organisms that predominate in human chorioamnionitis are important to allow for more faithful modeling of the disease process (Cappelletti et al., 2020).

### Feature 2: Primary Intrauterine Inflammation

Most models of acute chorioamnionitis do not involve an ascending route of spread but instead begin with systemic (intraperitoneal or subcutaneous injection) or direct intrauterine injection (Kramer et al., 2001; Kramer, 2011; Vlassaks et al., 2012; Lambermont et al., 2014; Di Simone et al., 2017). Models involving systemic inflammation are the closest approximations of preterm birth in response to maternal systemic inflammation, which occurs in cases of pyelonephritis and influenza. Caution should be used when generalizing findings from these models to the more common scenario of acute chorioamnionitis in which inflammation is greatest within the uterus, remains local, and occurs in the absence of clinical signs or symptoms in the pregnant mother (Gravett et al., 1986; Gibbs et al., 1992; Gomez-Lopez et al., 2018). For these reasons, intrauterine injection of inflammatory agents allows a closer recapitulation of acute chorioamnionitis that results from ascending spread. The technique of injection used depends on the size of the animal species. Injections in mice can be made into the uterine horn between amniotic sacs without entering the amniotic cavity (Rinaldi et al., 2015). Until recently, these injections were performed following mini-laparotomy (Hirsch et al., 1999), which itself was a confounding source of inflammation (Gomez-Lopez et al., 2018), but models involving ultrasound-guided injections have allowed the avoidance of a surgical procedure (Rinaldi et al., 2015; Gomez-Lopez et al., 2018). Based on evidence from human tissues showing that inflammation is greater in amniotic fluid than in fetal membranes, injection of agents directly into the amniotic fluid seems to be the most faithful model of the human chorioamnionitis scenario. This notion has been confirmed in studies in which the instillation of endotoxin into the uterine wall *versus* uterine cavity in pregnant sheep led to disparate outcomes (Newnham et al., 2002).

### Feature 3: Polymicrobial Infection, Biofilms, and Maternal Immune Modulation (Two-Hit Model and the Concept of Viral Priming)

Although infections underlying human acute chorioamnionitis are frequently polymicrobial and associated with biofilm formation, existing animal models of the condition have yet to recapitulate of these features. Biofilm formation is likely an important consideration for research focused on strategies for the eradication of infection (Kemp et al., 2014; Miura et al., 2014; Keelan, 2018). Recent work using viral inoculation prior to bacterial induction of chorioamnionitis in mice illustrates the potential of this system to advance understanding of a two-hit model of chorioamnionitis (Racicot et al., 2013; Cappelletti et al., 2017; Cross et al., 2017). While models incorporating the concept of viral priming are rare and mostly discussed using animal models, many models involve numerous sequential bacterial insults. This raises the possibility that the mechanism of disease induction depends not only on cumulative infection but also modulation of the maternal immune response (Snyder et al., 2013).

### Feature 5: Fetal Inflammatory Response and Preterm Birth

Fetal inflammatory response syndrome (FIRS) is (independent of preterm birth) associated with increased infant morbidity and multiple organ dysfunction (Kunzmann et al., 2010; Kuypers et al., 2013). Successful production of the fetal inflammatory response syndrome is therefore an important criterion for models aimed at studying the fetal response and its downstream consequences. FIRS can be confirmed by measuring IL-6 in fetal plasma and its incidence has been quantified for several models of acute chorioamnionitis (Rinaldi et al., 2015; Gomez-Lopez et al., 2018). Therapeutic interventions for FIRS using both rodent and non-rodent animal models have been summarized (Xiong and Wintermark, 2020).

As mentioned above, using animal models to decipher mechanisms of chorioamnionitis-induced preterm birth requires an anatomic system that closely resembles the placental and membrane architecture of human pregnancy. With their hemo-chorial placenta, rodent models are well suited for such studies, as are non-human primates. As seen in **Table 1**, numerous rodent models recapitulate the preterm birth phenomenon. Differences in fetal development at the time of birth between humans and rodents, however, preclude meaningful comparison with humans in terms of the consequences of preterm birth for long-term organ development.

## Discussion

Chorioamnionitis is a clinical syndrome with short and long-term clinical implications for maternal and fetal health. New strategies should focus on better prediction of chorioamnionitis and may include the use of omics (Ngo et al., 2018). Integrated trajectories of maternal metabolome, proteome, and immunome could define and characterize the patients at risk (Pique-Regi et al., 2019; Stelzer et al., 2021). Treatment strategies may include the modulation of maternal immune responses such as the use of extracellular vesicles (exosomes) or peptides (Spinelli et al., 2020). Furthermore, modulation of the immune dysregulation induced by the pro-inflammatory cascade should be considered and modulation of maternal vaginal microbiota is promising (Chougnet, 2018; Al-Nasiry et al., 2020). More attention is needed to investigate the efficacy of these prophylactic and therapeutic strategies. Together, clinical research efforts in chorioamnionitis are providing important and exciting clues but the selective and strategic use of animal models is necessary to understand the mechanistic basis of the key features of the disease. As summarized above, numerous key aspects of chorioamnionitis can be recapitulated using animal models. These models have great potential to lead to a better understanding of the pathophysiology of this syndrome and finally to the development of relevant and effective prophylactic/therapeutic approaches.

## Author Contributions

AB designed and wrote the manuscript and illustrations. SA-N wrote and edited the manuscript. MM and BK conceived the original idea, supervised the project and edited the manuscript. All authors contributed to the article and approved the submitted version.

## Conflict of Interest

The authors declare that the research was conducted in the absence of any commercial or financial relationships that could be construed as a potential conflict of interest.

## Publisher’s Note

All claims expressed in this article are solely those of the authors and do not necessarily represent those of their affiliated organizations, or those of the publisher, the editors and the reviewers. Any product that may be evaluated in this article, or claim that may be made by its manufacturer, is not guaranteed or endorsed by the publisher.
